# Implications of Serum IgG4 Levels for Pancreatobiliary Disorders and Cancer

**DOI:** 10.3390/jcm13133651

**Published:** 2024-06-22

**Authors:** Ching-Tang Tseng, Yi-Jun Liao, Cheng-Li Lin, Yen-Chun Peng

**Affiliations:** 1 Division of Gastroenterology and Hepatology, Department of Internal Medicine, Taichung Veterans General Hospital, Taichung 407, Taiwan; frank79503@hotmail.com (C.-T.T.); s19001029@gmail.com (Y.-J.L.); 2 Department of Post-Baccalaureate Medicine, School of Medicine, National Chung Hsing University, Taichung 402, Taiwan; 3 Management Office for Health Data, Clinical Trial Center (CTC), China Medical University Hospital, Taichung 404, Taiwan; orangechengli@gmail.com; 4 School of Medicine, National Yang Ming Chiao Tung University, Taipei 112, Taiwan

**Keywords:** IgG4 levels, pancreatobiliary, inflammatory diseases, malignancy

## Abstract

**Background/Objectives:** Immunoglobulin G4-related disease (IgG4-RD) is an immune-mediated disorder presenting as mass-like lesions with obstructions. An elevated serum IgG4 level is identified in more than half of affected patients and is considered a diagnostic criterion. IgG4-RD is still easily misdiagnosed as neoplastic or infectious disease. We aimed to conduct a hospital-based study to illuminate the association between serum IgG4 levels and pancreatobiliary disorders and cancer. **Methods**: In this study, serum IgG4 levels were assessed at our hospital’s immunology laboratory, utilizing data from the hospital’s computer center, and the diagnostic codes used were based on ICD-9-CM. We analyzed IgG4 level data collected between April 2013 and April 2020, including patients’ age, gender, and diseases, but excluding the rationale for IgG4 level assessment. Employing propensity score matching (PSM) at a 1:1 ratio to mitigate age and gender confounding, we analyzed 759 patients divided into groups by IgG4 levels (≤140 and >140 mg/dL; and ≤140, 141–280, >280 mg/dL). We explored associations between IgG4 levels and conditions such as pancreatobiliary cancer (the group included cholangiocarcinoma, pancreatic cancer, and ampullary cancer), cholangitis, cholangiocarcinoma, pancreatitis, pancreatic cancer, and ampullary cancer. **Results**: Our study analyzed the demographics, characteristics, and serum IgG4 levels of participants and found no significant differences in serum IgG4 levels across various pancreatobiliary conditions. Nevertheless, the crude odds ratios (ORs) suggested a nuanced association between a higher IgG4 level > 280 mg/dL and increased risks of cancer and pancreatitis, with crude ORs of 1.52 (*p* = 0.03) and 1.49 (*p* = 0.008), respectively. After PSM matching, the further analysis of 759 matched patients showed no significant differences in IgG4 levels > 140 mg/dL between cancerous and non-cancerous groups, nor across other pancreatobiliary conditions. A higher serum IgG4 level > 280 mg/dL was significantly associated with pancreatobiliary cancer and cholangiocarcinoma, with crude ORs of 1.61 (*p* = 0.026) and 1.62 (*p* = 0.044), respectively. In addition, IgG4 > 280 mg/dL showed a greater association with pancreatic cancer compared with 141–280 mg/dL, with crude OR of 2.18 (*p* = 0.038). **Conclusions**: Our study did not find a clear association between serum IgG4 levels (>140 mg/dL) and pancreatobiliary cancer. We observed that higher IgG4 levels (>280 mg/dL) may be associated with cholangiocarcinoma and pancreatic cancer, as indicated by crude ORs. However, the adjusted analysis did not demonstrate the significant association between IgG4 level > 280 mg/dL and cancer. Considering IgG4-RD as a chronic and persistent inflammatory status, it is more closely associated with inflammatory diseases than with cancer. Therefore, further long-term cohort studies are necessary to evaluate the potential role of IgG4 levels in cancer risk among these patients.

## 1. Introduction

IgG4 is the latest defined human IgG subclass in serum and an intriguing antibody with unique biological properties [1]. Clinically, IgG4-related disease (IgG4-RD) involves a chronic fibrous inflammatory process related to immunomodulation disorder, characterized as tumor-like lesions with dense lymphoplasmacytic infiltration with IgG4-positive plasma cells, and the most affected organs are the pancreas, bile duct, major salivary glands, lacrimal glands, retroperitoneum, kidneys, and lymphatic ducts. In addition to local inflammation-induced tissue fibrosis and injury, IgG4-RD has been recognized as a systemic disorder and was found to be linked to elevated serum IgG4 levels. As it is a newly defined disease, the global incidence and prevalence of IgG4-RD is underestimated. In addition, the natural history of IgG4-RD is also limited due its chronic nature and fluctuating clinical course [2,3,4,5,6]. The trigger of IgG4 production is thought to involve the activation of the innate immune response by microbe- and/or damage-associated molecular patterns that stimulate the production of the type I interferon and B cell-activating factor by innate immune cells, resulting in IgG4 production by B cells [7]. Thus, the serum IgG4 level is considered an important indicator for suspicion of the diagnosis of IgG4-RD.

Cancer is a multifactorial and complex disease process caused by cells that have lost their physiological control of cell overgrowth [8]. Chronic infection results in persistent stimulation and inflammation and, thus, is one of the main contributors to cancer development [9,10]. Most previous studies were related to the association of cancer concomitant with IgG4-RD [11,12]. Cancer could trigger autoantigen expression, leading to IgG4-RD, and an increased risk of IgG4-RD resulting from cancer treatment has also been proposed [13]. A recent multi-hospital study enrolled 121 patients and demonstrated that IgG4-sclerosing cholangitis carried potential risk for cancer [14]. There is also a lack of basic research on the molecular mechanism of IgG4 and carcinogenesis. A study of increased IgG4-containing lymphocytes in esophageal cancer implicated the role of IgG4 in the cancer micro-environment. The authors reported that IgG4 could play a role in cancer treatment [15].

The main histopathological features of IgG4-RD include a dense, polyclonal, lymphoplasmacytic infiltrate with an abundance of IgG4-positive plasma cells, storiform fibrosis, and obliterative phlebitis. These manifestations can range from mild to severe, and the natural history is wide-ranging and variable. The precise pathogenic mechanisms of IgG4-RD and its potential association with cancer risk remain unclear and require further investigation [6]. There are possible malignancies in patients with IgG4-RD at the time of diagnosis and follow-up in the natural course [16]. Thus, the differential diagnosis of IgG4-RD versus cancer with serum IgG4 level elevation may be difficult in some situations. 

There is a lack of data showing the associations between IgG4-RD, serum IgG4 level, and cancer risk, particularly pancreatic, biliary, and ampullary cancers. Hence, we investigated the associations of serum IgG4 level with various cancers. We were interested in determining whether serum IgG4 level was associated with cancer and whether different serum levels were associated with a higher risk of cancer. Furthermore, we attempted to explore the association of a high serum IgG4 level, which indicates more definitive IgG4-RD, with carcinogenesis. While there is scanty clinical evidence, IgG4-RD could be associated with malignancy; IgG4-RD may present with a tumor-like focal tumefactive lesion, which could lead to misdiagnosis [17]. Thus, the evidence and risk of carcinogenesis related to IgG4-RD need to be clarified.

To address these questions, we conducted a hospital-based case–control study to analyze the association of IgG4 serum level among patients with cancer.

## 2. Materials and Methods

### 2.1. Data Source

Patients’ serum IgG4 levels were checked at the laboratory of the immunology division in our hospital. The data were obtained from the database of our hospital computer center. The diagnostic codes used were based on the International Classification of Diseases, 9th Revision, Clinical Modification (ICD-9-CM). This study was approved by the Institutional Review Board of our institution (Taichung Veterans General Hospital IRB: CE20314B).

### 2.2. Subjects

**Figure 1:**
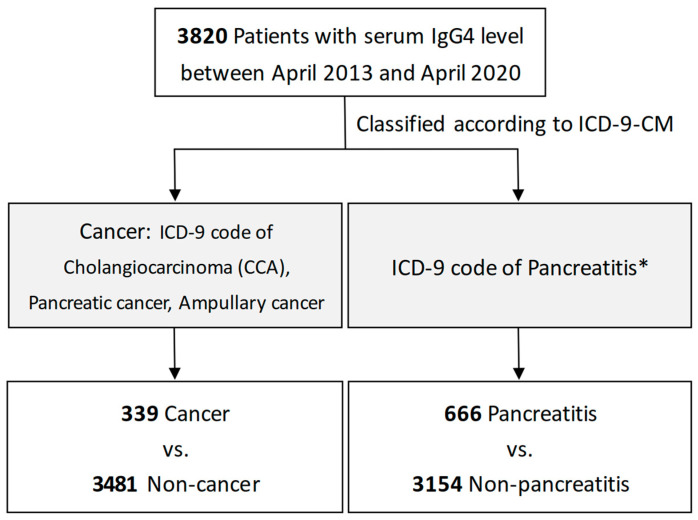
Flowchart for patients with cancer or pancreatitis. * Discusses pancreatobiliary disorders focusing on pancreatitis, including acute and chronic pancreatitis.

During April 2013 to April 2020, we collected data on serum IgG4 levels from our hospital database. Other data included the patients’ age, gender, and diseases. The reason for checking the serum IgG4 level was according to the clinical physicians’ judgment, and there was no prior record in the database. The diagnosis of cancer and pancreatobiliary disorders were determined using diagnostic codes based on ICD-9-CM (Figure 1). Cancer included cholangiocarcinoma (CCA), pancreatic cancer, and ampullary cancer. In pancreatobiliary disorders, we mainly focused on pancreatitis, including acute and chronic pancreatitis.

#### Definition of Pancreatobiliary Disorders

The diagnosis of cholangitis was made by Tokyo guidelines 2013/2018, including systemic inflammation, cholestasis, and imaging implying infection and biliary obstruction [18,19,20]. The diagnosis of acute pancreatitis required the presence of two of the following three criteria: the acute onset of persistent, severe, epigastric pain often radiating to the back; an elevation in serum lipase or amylase to three times or greater than the upper limit of normal; and characteristic findings of acute pancreatitis on imaging [21]. On the other hand, the diagnosis of chronic pancreatitis was based on exposure risk; underlying predisposition; clinical presentation, including chronic abdominal pain and/or a history of relapsing acute pancreatitis; symptoms of pancreatic exocrine insufficiency (diarrhea, steatorrhea, or weight loss); or pancreatogenic diabetes, and using other modalities, including cross-sectional imaging, and pancreatic function tests [22,23]. We linked the above diagnoses with ICD-9-CM.

### 2.3. The Associations between Serum IgG4 Levels and the Risk of Different Outcomes by Propensity Score Matching

**Figure 2:**
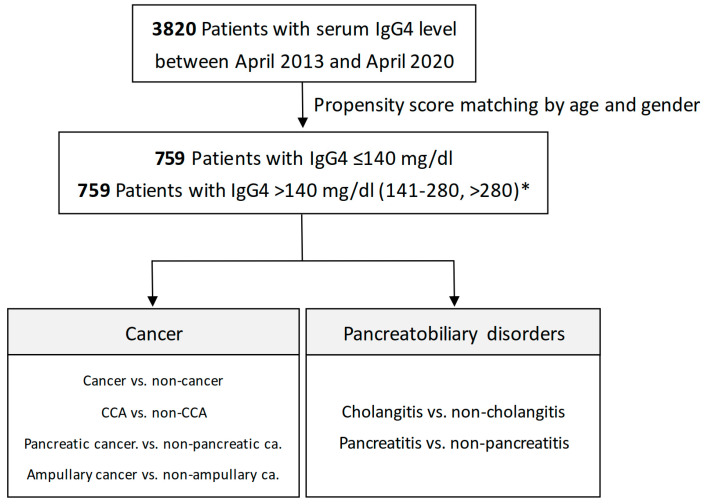
Flowchart for serum IgG4 levels and different outcomes after propensity score matching (PSM). * Serum IgG4 levels were divided into different groups, i.e., ≤140, 141–280, and >280 mg/dL.

To address the confounding effects of age and gender in our analysis, we employed propensity score matching (PSM) at a 1:1 ratio (Figure 2). This approach resulted in the selection of 759 patients, categorized into two groups based on their serum IgG4 levels: those with IgG4 ≤ 140 mg/dL and those with IgG4 > 140 mg/dL. These groups were then analyzed to investigate associations between serum IgG4 levels and various conditions, including cancer versus non-cancer, cholangitis versus non-cholangitis, cholangiocarcinoma versus non-cholangiocarcinoma, pancreatitis versus non-pancreatitis, pancreatic cancer versus non-pancreatic cancer, and ampullary cancer versus non-ampullary cancer, to facilitate a more detailed exploration of the relationships between serum IgG4 levels and these conditions. For further determination of the significant role in serum IgG4 levels in pancreatic and biliary diseases, we divided serum IgG4 levels into different groups, i.e., ≤140, 141–280, and >280 mg/dL, and determined the associations with pancreatic and biliary diseases, according to the International Consensus Diagnostic Criteria (ICDC), which indicated that the marked elevation of serum IgG4 (>2 times upper limit of normal; >280 mg/dL in our study) is strongly suggestive (Level 1 criteria) of autoimmune pancreatitis (AIP) in the setting of obstructive jaundice/pancreatic mass, and the serum IgG4 level between 141 and 280 mg/dL is Level 2 criteria [24].

### 2.4. Statistical Analysis

The statistical analyses were performed as follows: the Chi-square test was used to check the differences in categorical variables between the cancer and non-cancer groups, while the two-sample Student’s *t*-test was used to examine continuous variables. Univariable and multivariable logistic regression were used to estimate the effect of serum IgG4 levels on the risk of cancer, as indicated by the odds ratio (OR) with a 95% confidence interval (CI). All analyses were performed using SAS statistical software (version 9.4; SAS Institute, Inc., Cary, NC, USA), and results were considered statistically significant when two-tailed *p*-values were less than 0.05.

## 3. Results

### 3.1. Demographics and Characteristics of Study Subjects 

In Table 1, there was a higher proportion of females in the cancer group compared to the non-cancer group (57.82% vs. 38.50%, *p* < 0.001). Furthermore, the mean age of individuals in the cancer group was 63.81 (±14.19) years, which was considerably higher than the mean age of 53.3 (±16.69) years in the non-cancer control group and showed a statistically significant difference (*p* < 0.001). In terms of serum IgG4 levels (categorized as ≤140, 141–280, and >280 mg/dL), a notable disparity was found between cancer and non-cancer patients, accounting for 80.24%, 12.90%, and 6.87% of patients in the non-cancer group and 74.93%, 15.34%, and 9.73% in the cancer group, which was statistically significant (*p* < 0.05). These findings provide valuable insights into the differences between cancer and non-cancer patients across various demographic and IgG4 measures. A higher proportion of cancer patients had higher serum IgG4 levels compared with non-cancer patients.

### 3.2. Risk of Cancer Associated with Serum Level of IgG4 

Table 2 displays the odds ratios (ORs) representing the estimated cancer risk associated with different IgG4 levels (≤140, 141–280, and >280 mg/dL). In the crude analysis, the ORs for IgG4 levels 141–280 mg/dL and >280 mg/dL were 1.27 (95% CI: 0.93–1.74, *p* = 0.13) and 1.52 (95% CI: 1.03–2.23, *p* = 0.03), respectively. Following adjustment for age and gender, the adjusted ORs for cancer risk in the IgG4 141–280 mg/dL and IgG4 > 280 mg/dL categories were 1.20 and 1.17, with 95% confidence intervals of 0.87–1.66 and 0.78–1.73, respectively. Interestingly, it was observed that cancer patients exhibited significantly higher IgG4 levels compared to non-cancer patients based on the crude OR analysis. These findings point to a potential association between higher IgG4 levels and increased cancer risk, particularly in the higher IgG4 category.

### 3.3. Odds of Pancreatitis Associated with Serum Level of IgG4

In our investigation, we aimed to elucidate the relationship between serum IgG4 levels and the incidence of pancreatitis. Our objective was to quantify the association between different concentrations of IgG4 (≤140, 141–280, and >280 mg/dL) and the likelihood of developing pancreatitis. According to Table 3, the preliminary analysis yielded odds ratios (ORs) of 0.92 (95% CI: 0.71–1.19, *p* = 0.52) for IgG4 levels between 141 and 280 mg/dL and 1.49 (95% CI: 1.11–2.00, *p* = 0.008) for levels above 280 mg/dL. Adjusting for age and gender, the adjusted ORs for IgG4 levels in the 141–280 mg/dL and >280 mg/dL categories were 0.85 and 1.21, with 95% confidence intervals of 0.66–1.11 and 0.89–1.64, respectively. These findings suggest a nuanced link between increased IgG4 levels and a heightened risk of pancreatitis, especially at higher IgG4 concentrations.

### 3.4. Serum IgG4 Level >140 mg/dL in Different Pancreatobiliary Patient Groups after Propensity Score Matching

Following propensity score matching (PSM) with a 1:1 ratio, we identified 759 patients with IgG4 ≤ 140 mg/dL and another 759 with serum IgG4 levels > 140 mg/dL for in-depth analysis (Figure 2; Supplementary Table S1 shows the baseline characteristics after PSM). In Table 4, our findings indicate that IgG4 levels did not significantly differ between cancer patients and their non-cancer counterparts, with a crude OR of 1.20 (95% CI: 0.86–1.67, *p* = 0.276) and an adjusted OR of 1.21 (95% CI: 0.86–1.69, *p* = 0.266).

Similarly, for patients with cholangitis, the IgG4 levels were not significantly different from those without the condition, with both crude and adjusted ORs of 1.07 (95% CI: 0.80–1.44, *p* = 0.652; and 0.79–1.45, *p* = 0.644, respectively). The analysis of cholangiocarcinoma (CCA) patients also revealed no significant difference in IgG4 levels compared to non-CCA patients, with both crude and adjusted ORs of 1.18 (95% CI: 0.81–1.72, *p* = 0.391; and 0.81–1.73, *p* = 0.383, respectively).

The investigation of pancreatitis patients demonstrated no significant elevation in IgG4 levels compared to non-pancreatitis individuals, with ORs of 0.85 (95% CI: 0.66–1.09, *p* = 0.202) in the crude analysis and 0.84 (95% CI: 0.65–1.09, *p* = 0.193) upon adjustment. The assessment of pancreatic cancer patients also showed no significant difference in IgG4 levels, with both crude and adjusted ORs of 1.12 (95% CI: 0.66–1.90, *p* = 0.686; and 0.66–1.91, *p* = 0.683, respectively). Lastly, the analysis of ampullary cancer patients found no significant variation in IgG4 levels, with both crude and adjusted ORs of 1.00 (95% CI: 0.39–2.53, *p* = 1; and 0.39–2.54, *p* = 1, respectively).

### 3.5. The Association of Higher Serum IgG4 Level >280 mg/dL with Pancreatic and Biliary Diseases

Following propensity score matching (PSM) with a 1:1 ratio, we further determined the association of different IgG4 levels (≤140, 141–280, and >280 mg/dL) with pancreatic and biliary diseases (Figure 2). In Table 5, our findings indicate that IgG4 level > 280 mg/dL was significantly associated with cancer and cholangiocarcinoma, with crude ORs of 1.61 (95% CI: 1.06–2.44, *p* = 0.026) and 1.62 (95% CI: 1.01–2.59, *p* = 0.044), respectively. For pancreatic cancer, IgG4 > 280 mg/dL showed a greater association with pancreatic cancer compared with 141–280 mg/dL, with crude OR of 2.18 (95% CI: 1.05–4.53, *p* = 0.038). There was no significant correlation with IgG4 > 280 mg/dL in ampullary cancer compared with ≤140 mg/dL and 141–280 mg/dL, with crude ORs of 0.63 (95% CI: 0.14–2.93, *p* = 0.555) and 0.52 (95% CI: 0.11–2.54, *p* = 0.421), respectively.

For cholangitis, IgG4 > 280 mg/dL was also significantly correlated, with a crude OR of 1.50 (95% CI: 1.03–2.18, *p* = 0.035). For pancreatitis, IgG4 > 280 mg/dL showed a greater association with pancreatitis compared with 141–280 mg/dL, with a crude OR of 1.81 (95% CI: 1.25–2.61, *p* = 0.002) and an adjusted OR of 1.54 (95% CI: 1.05–2.25, *p* = 0.027).

## 4. Discussion

Our analysis revealed that IgG4 levels showed distinct features among patients diagnosed with cholangitis, pancreatitis, and biliary–pancreatic–ampullary cancer. Markedly elevated serum IgG4 levels (>280 mg/dL) were found in patients undergoing IgG4 testing for other reasons for cholangiocarcinoma and pancreatic cancer, but not ampullary cancer. The analysis of biliary–pancreatic–ampullary cancer risk in relation to IgG4 levels showed a notably significant crude odds ratio (OR) for patients with higher IgG4 levels (>280 mg/dL). However, this significance lessened after accounting for age and gender, suggesting that the initial observed relationship was influenced by these demographic factors. These findings underscore potential connections between specific demographic and clinical characteristics in patients with biliary, pancreatic, or ampullary cancer and the levels of IgG4.

In clinical practice, it may be unclear whether patients with biliary or pancreatic obstructive diseases have cancer or not. Patients with IgG4-RD could just have a concomitantly elevated serum IgG4 level or they may indeed have an occult cancer component. The challenge for the physician is to assess the risk or odds of cancer, particularly biliary or pancreatic cancer, in patients with elevated serum IgG4 levels. Our results demonstrated that the association of serum IgG4 levels with biliary–pancreatic–ampullary cancer was not significant. These results may help physicians to make decisions for patients with obstructive disease and elevated serum IgG4 levels. A study has demonstrated the increasing clinical importance of both autoimmune pancreatitis and cholangitis, which can mimic pancreatic cancer and biliary cancer. IgG4 levels in tissue or blood cannot be used alone to diagnose IgG4-RD, and they should be adjunct to clinical, radiological, and histological features. There is importance in the differential diagnosis of inflammation and cancer, resulting in a significant impact on the clinical management of affected patients [25].

For diagnosing AIP, the International Association of Pancreatology proposed the ICDC, which combined pancreatic parenchyma and ductal changes at abdominal imaging, the serum IgG4 level, other organ involvement, histology, and the response to steroid treatment to reach a diagnosis [24]. Endoscopic ultrasound (EUS) and EUS-guided fine-needle aspiration/biopsy (EUS-FNA/B) were the tissue-acquisition techniques most used for AIP. In the clinical practice of one cohort study, endoscopic cytohistology was available in 46.2% of cases, and diagnostics for AIP was available in 35.2%. EUS-FNA/B of the pancreas was conducted mostly in the focal form of AIP (85% of cases) to exclude pancreatic cancer [26].

IgG4 may contribute to tumor-associated escape from immune surveillance and have implications for cancer immunotherapy [16,27]. In a case report, lung tumor with lung IgG4 interstitial infiltration was observed [11]. However, there are no large series of cases or surveys of IgG4 tumors in patients with lung disease in the literature. It has been reported that there is a potential for lymphoma to develop in patients with IgG4-RD. The co-occurrence of IgG4-RD and lymphoma has also been reported [28]. In another study, a negative association between IgG4 expression and cutaneous marginal zone lymphoma was found [29]. Regarding immunity, IgG4 antibodies and IgG4+ B cells in different cancers could involve IgG4 in tumor escape from immune surveillance through a number of potential mechanisms, including the IgG4 blockade of IgG1-mediated effector functions [15,27]. A proposed mechanism by which IgG4 may contribute to tumor-associated escape from human immune surveillance involves the possibility that inflammatory conditions may exist in tumors that support IgG4 [27]. IgG4 in inflammatory and cancer conditions is thus complex and sometimes confusing. As such, elevated IgG4 levels could result in malignancies being overlooked or misdiagnosed.

In an earlier survey of a general population for malignancies in IgG4-RD, malignancies were observed in 10.4% of the IgG4-RD patients. The malignancies were all different and included lung cancer, colon cancer, and lymphoma [16]. Except for age at the time the IgG4-RD diagnosis was made, there were no common features in patients with cancer. The frequency of history of malignancy was >3-fold higher in IgG4-RD patients, according to an analysis of an epidemiological database [13]. By timeframe, the results revealed that a history of malignancy was linked with a risk of IgG4-RD. There is a lack of evidence showing any causal relationships between IgG4-RD and malignancy.

In pancreatobiliary type IgG4-RD, there was a greater risk of developing cancer [14,16]. In the aforementioned multiple-hospital-based retrospective study, the authors concluded that IgG4-sclerosing cholangitis patients had a high risk of pancreatic and bile duct cancer. The risk of cancer was high <1 year and >5 years after the diagnosis of IgG4-sclerosing cholangitis. The higher risk at less than 1 year may have been due to the diagnosis of IgG4-RD and cancer at the same time. The present study found that a mildly elevated IgG4 level (>140 mg/dL) was not associated with pancreatobiliary disorders and cancer. A marked elevation of IgG4 levels (>280 mg/dL) was nuanced and associated with cholangiocarcinoma and pancreatic cancer, but not ampullary cancer.

The main strengths of this hospital-based study were the use of a validated database and disease diagnoses. There were some limitations in this study. First, the associations of patients’ serum IgG4 levels with diagnoses of cancer were based solely on claims data. There was no definitive diagnosis of IgG4-RD by pathological proof in the analysis of associations with cancer. Second, as this was a case–control study, we did not determine the causal relationship between serum IgG4 level and cancer. Third, the treatments of both IgG4-RD and cancer were not included in this study.

## 5. Conclusions

Our study did not find a clear association between serum IgG4 levels (>140 mg/dL) and pancreatobiliary cancer. We observed that higher serum IgG4 levels (>280 mg/dL) may be associated with cholangiocarcinoma and pancreatic cancer, as indicated by crude odds ratios (ORs). However, the adjusted analysis did not demonstrate the significant association between IgG4 level > 280 mg/dL and cancer. Considering IgG4-RD as a chronic and persistent inflammatory status, it is more closely associated with inflammatory diseases than with cancer. Therefore, further long-term cohort studies are necessary to evaluate the potential role of IgG4 levels in cancer risk among these patients.

## Figures and Tables

**Table 1:** Baseline characteristics of serum IgG4 levels in cancer and non-cancer patients.

Variable	Non-Cancer (N = 3481)		Cancer ^2^ (N = 339)		
	N	%	N	%	*p*-Value
Sex					<0.0001
Female	1340	38.50	196	57.82	
Male	2141	61.51	143	42.18	
Age (years)					<0.0001
0–39	776	22.29	16	4.72	
40–59	1390	39.93	110	32.45	
60–79	1146	32.92	173	51.03	
>79	169	4.86	40	11.80	
Mean, (SD) ^1^	53.3	(16.69)	63.81	(14.19)	<0.0001
IgG4 value (mg/dL)					0.048
≤140	2793	80.24	254	74.93	
141–280	449	12.90	52	15.34	
>280	239	6.87	33	9.73	
Mean, (SD) ^1^	118.5	(223)	173.95	(483.31)	0.038

^1^ Student’s *t*-test; ^2^ cancer: cholangiocarcinoma, pancreatic cancer, ampullary cancer. Abbreviations: N, numbers of patients; SD, standard deviation.

**Table 2:** Odds ratios of cancer.

	Non-Cancer		Cancer ^1^		Crude			Adjusted		
Variable	N	%	N	%	cOR (95% CI)		*p*-Value	aOR ^†^ (95% CI)		*p*-Value
IgG4 value (mg/dL)										
≤140	2793	80.24	254	74.93	1.00	(reference)	-	1.00	(reference)	-
141–280	449	12.90	52	15.34	1.27	(0.93, 1.74)	0.13	1.20	(0.87, 1.66)	0.26
>280	239	6.87	33	9.73	1.52	(1.03, 2.23) *	0.03	1.17	(0.78, 1.73)	0.45

^†^ Adjusted by age and gender; * *p* < 0.05. ^1^ Cancer: cholangiocarcinoma, pancreatic cancer, ampullary cancer. Abbreviations: N, numbers of patients; cOR, crude odds ratio; aOR, adjusted odds ratio; CI, confidence interval.

**Table 3:** Odds ratios of pancreatitis.

	Non-Pancreatitis		Pancreatitis		Crude			Adjusted		
Variable	N	%	N	%	cOR (95% CI)		*p*-Value	aOR ^†^ (95% CI)		*p*-Value
IgG4 value (mg/dL)										
≤140	2525	80.06	522	78.38	1.00	(reference)	-	1.00	(reference)	-
141–280	421	13.35	80	12.01	0.92	(0.71, 1.19)	0.52	0.85	(0.66, 1.11)	0.24
>280	208	6.59	64	9.61	1.49	(1.11, 2.00) **	0.008	1.21	(0.89, 1.64)	0.23

^†^ Adjusted by age and gender; ** *p* < 0.01. Abbreviations: N, numbers of patients; cOR, crude odds ratio; aOR, adjusted odds ratio; CI, confidence interval.

**Table 4:** The associations between IgG4 values (≤140 and >140 mg/dL) and risks of different outcomes, after propensity score matching (a matching ratio of 1:1).

	Non-Outcome		Outcome		Crude			Adjusted		
Variable	N	%	N	%	OR (95% CI)		*p*-Value	OR ^†^ (95% CI)		*p*-Value
	Cancer									
IgG4 value (mg/dL)										
≤140	686	50.48	73	45.91	1.00	(reference)	-	1.00	(reference)	-
>140	673	49.52	86	54.09	1.20	(0.86, 1.67)	0.276	1.21	(0.86, 1.69)	0.266
Sex										
Female	632	46.50	42	26.42	1.00	(reference)	-	1.00	(reference)	-
Male	727	53.50	117	73.58	2.42	(1.68, 3.50) ***	<0.001	2.09	(1.44, 3.05) ***	<0.001
Age (years)										
18–50	492	36.20	16	10.06	1.00	(reference)	-	1.00	(reference)	-
51–64	400	29.43	62	38.99	4.77	(2.71, 8.39) ***	<0.001	4.50	(2.55, 7.95) ***	<0.001
65–92	467	34.36	81	50.94	5.33	(3.07, 9.25) ***	<0.001	4.65	(2.67, 8.11) ***	<0.001
	Cholangitis									
IgG4 value (mg/dL)										
≤140	660	50.23	99	48.53	1.00	(reference)	-	1.00	(reference)	-
>140	654	49.77	105	51.47	1.07	(0.80, 1.44)	0.652	1.07	(0.79, 1.45)	0.644
Sex										
Female	618	47.03	56	27.45	1.00	(reference)	-	1.00	(reference)	-
Male	696	52.97	148	72.55	2.35	(1.69, 3.25) ***	<0.001	2.01	(1.44, 2.80) ***	<0.001
Age (years)										
18–50	480	36.53	28	13.73	1.00	(reference)	-	1.00	(reference)	-
51–64	395	30.06	67	32.84	2.91	(1.83, 4.61) ***	<0.001	2.73	(1.72, 4.34) ***	<0.001
65–92	439	33.41	109	53.43	4.26	(2.76, 6.58) ***	<0.001	3.71	(2.39, 5.77) ***	<0.001
	Cholangiocarcinoma									
IgG4 value (mg/dL)										
≤140	704	50.32	55	46.22	1.00	(reference)	-	1.00	(reference)	-
>140	695	49.68	64	53.78	1.18	(0.81, 1.72)	0.391	1.18	(0.81, 1.73)	0.383
Sex										
Female	640	45.75	34	28.57	1.00	(reference)	-	1.00	(reference)	-
Male	759	54.25	85	71.43	2.11	(1.40, 3.18) ***	<0.001	1.78	(1.17, 2.72) **	0.007
Age (years)										
18–50	498	35.6	10	8.4	1.00	(reference)	-	1.00	(reference)	-
51–64	415	29.66	47	39.5	5.64	(2.82, 11.30) ***	<0.001	5.33	(2.66, 10.71) ***	<0.001
65–92	486	34.74	62	52.1	6.35	(3.22, 12.53) ***	<0.001	5.62	(2.84, 11.15) ***	<0.001
	Pancreatitis									
IgG4 value (mg/dL)										
≤140	595	49.17	164	53.25	1.00	(reference)	-	1.00	(reference)	-
>140	615	50.83	144	46.75	0.85	(0.66, 1.09)	0.202	0.84	(0.65, 1.09)	0.193
Sex										
Female	593	49.01	81	26.30	1.00	(reference)	-	1.00	(reference)	-
Male	617	50.99	227	73.70	2.69	(2.04, 3.56) ***	<0.001	2.50	(1.89, 3.32) ***	<0.001
Age (years)										
18–50	430	35.54	78	25.32	1.00	(reference)	-	1.00	(reference)	-
51–64	366	30.25	96	31.17	1.45	(1.04, 2.01) *	0.028	1.33	(0.95, 1.86)	0.096
65–92	414	34.21	134	43.51	1.78	(1.31, 2.43) ***	<0.001	1.49	(1.08, 2.05) *	0.014
	Pancreatic cancer									
IgG4 value (mg/dL)										
≤140	732	50.10	27	47.37	1.00	(reference)	-	1.00	(reference)	-
>140	729	49.90	30	52.63	1.12	(0.66,1.90)	0.686	1.12	(0.66,1.91)	0.683
Sex										
Female	662	45.31	12	21.05	1.00	(reference)	-	1.00	(reference)	-
Male	799	54.69	45	78.95	3.11	(1.63, 5.92) ***	<0.001	2.72	(1.41, 5.23) **	0.003
Age (years)										
18–50	501	34.29	7	12.28	1.00	(reference)	-	1.00	(reference)	-
51–64	438	29.98	24	42.11	3.92	(1.67, 9.19) **	0.002	3.54	(1.50, 8.34) **	0.004
65–92	522	35.73	26	45.61	3.56	(1.53, 8.29) **	0.003	2.90	(1.24, 6.79) *	0.014
	Ampullary cancer									
IgG4 value (mg/dL)										
≤140	750	50.00	9	50.00	1.00	(reference)	-	1.00	(reference)	-
>140	750	50.00	9	50.00	1.00	(0.39, 2.53)	1	1.00	(0.39, 2.54)	1
Sex										
Female	668	44.53	6	33.33	1.00	(reference)	-	1.00	(reference)	-
Male	832	55.47	12	66.67	1.61	(0.60, 4.30)	0.346	1.30	(0.48, 3.53)	0.612
Age (years)										
18–50	506	33.73	2	11.11	1.00	(reference)	-	1.00	(reference)	-
51–64	460	30.67	2	11.11	1.10	(0.15, 7.84)	0.924	1.12	(0.16, 8.03)	0.907
65–92	534	35.6	14	77.78	6.63	(1.50, 29.33) *	0.013	6.70	(1.50, 30.02) *	0.013

Abbreviations: N, numbers of patients; OR, odds ratio; CI, confidence interval. ^†^ Adjusted OR: multivariable analysis including age and gender; * *p*-value < 0.05, ** *p* < 0.01, *** *p* < 0.001.

**Table 5:** The associations between IgG4 values (≤140, 141–280, and >280 mg/dL) and risks of different outcomes after propensity score matching (a matching ratio of 1:1).

	Non-Outcome		Outcome		Crude			Adjusted			Crude			**Adjusted**		
IgG4 Value (mg/dL)	N	%	N	%	OR (95% CI)		*p*-Value	OR ^†^ (95% CI)		*p*-Value	OR (95% CI)		*p*-Value	OR ^†^ (95% CI)		*p*-Value
	Cancer															
≤140	686	50.48	73	45.91	1.00	(reference)	-	1.00	(reference)	-						
141–280	445	32.74	47	29.56	0.99	(0.68, 1.46)	0.970	1.09	(0.74, 1.62)	0.656	1.00	(reference)	-	1.00	(reference)	-
>280	228	16.78	39	24.53	1.61	(1.06, 2.44) *	0.026	1.39	(0.91, 2.13)	0.128	1.62	(1.03, 2.55) *	0.037	1.20	(0.75, 1.93)	0.444
	Cholangitis															
≤140	660	50.23	99	48.53	1.00	(reference)	-	1.00	(reference)	-						
141–280	436	33.18	56	27.45	0.86	(0.60, 1.21)	0.384	0.93	(0.65, 1.33)	0.691	1.00	(reference)	-	1.00	(reference)	-
>280	218	16.59	49	24.02	1.50	(1.03, 2.18) *	0.035	1.31	(0.89, 1.93)	0.168	1.75	(1.15, 2.65) **	0.008	1.40	(0.91, 2.16)	0.122
	Cholangiocarcinoma															
≤140	704	50.32	55	46.22	1.00	(reference)	-	1.00	(reference)	-						
141–280	458	32.74	34	28.57	0.95	(0.61, 1.48)	0.821	1.04	(0.66, 1.63)	0.868	1.00	(reference)	-	1.00	(reference)	-
>280	237	16.94	30	25.21	1.62	(1.01, 2.59) *	0.044	1.41	(0.88, 2.28)	0.157	1.71	(1.02, 2.85) *	0.042	1.31	(0.77, 2.24)	0.314
	Pancreatitis															
≤140	595	49.17	164	53.25	1.00	(reference)	-	1.00	(reference)	-						
141–280	415	34.3	77	25	0.67	(0.50, 0.91) **	0.009	0.71	(0.52, 0.96) *	0.027	1.00	(reference)	-	1.00	(reference)	-
>280	200	16.53	67	21.75	1.22	(0.88, 1.68)	0.241	1.09	(0.78, 1.52)	0.614	1.81	(1.25, 2.61) **	0.002	1.54	(1.05, 2.25) *	0.027
	Pancreatic cancer															
≤140	732	50.10	27	47.37	1.00	(reference)	-	1.00	(reference)	-						
141–280	478	32.72	14	24.56	0.79	(0.41, 1.53)	0.491	0.87	(0.45, 1.69)	0.682	1.00	(reference)	-	1.00	(reference)	-
>280	251	17.18	16	28.07	1.73	(0.92, 3.26)	0.091	1.49	(0.78, 2.83)	0.224	2.18	(1.05, 4.53) *	0.038	1.66	(0.78, 3.52)	0.187
	Ampullary cancer															
≤140	750	50.00	9	50.00	1.00	(reference)	-	1.00	(reference)	-						
141–280	485	32.33	7	38.89	1.20	(0.44, 3.25)	0.716	1.31	(0.48, 3.56)	0.598	1.00	(reference)	-	1.00	(reference)	-
>280	265	17.67	2	11.11	0.63	(0.14, 2.93)	0.555	0.55	(0.12, 2.57)	0.444	0.52	(0.11, 2.54)	0.421	0.37	(0.08, 1.82)	0.221

Abbreviations: N, numbers of patients; OR, odds ratio; CI, confidence interval. ^†^ Adjusted OR: multivariable analysis including age and gender; * *p*-value < 0.05, ** *p* < 0.01.

## Data Availability

The data presented in this study are available on request from the corresponding author.

## Supplementary Materials

The following supporting information can be downloaded at https://www.mdpi.com/article/10.3390/jcm13133651/s1: Table S1: Baseline characteristics after propensity score matching by age and gender.
