# Supplementary material for: Implications of Serum IgG4 Levels for Pancreatobiliary Disorders and Cancer
Source: J Clin Med. 2024 Jun 22;13(13):3651. doi: 10.3390/jcm13133651 (PMC11242838; doi:10.3390/jcm13133651)
Supplement: Supplementary file 1 [file jcm-13-03651-s001.zip › jcm-2964635-supplementary.pdf]

Supplementary Table S1.

Table S1. Baseline characteristics after propensity score matching by age and gender

**Variable**	**IgG4 value ≤140 (N = 759)**		**IgG4 value >140 (N = 759)**		***p*-Value**
	**N**	**%**	**N**	**%**	
IgG4 value (mg/dL)					-
141–280	-	-	492	64.82	
>280	-	-	267	35.18	
Sex					>0.999
Female	337	44.4	337	44.4	
Male	422	55.6	422	55.6	
Age (years)					>0.999
18-50	254	33.47	254	33.47	
51-64	231	30.43	231	30.43	
65-92	274	36.1	274	36.1	
Mean, (SD) [1]	56.39	16.77	56.4	16.83	0.999

[1] Student's *t*-test; Abbreviations: N, numbers of patients; SD, standard deviation.
